# Closed-Loop Transcranial Ultrasound Stimulation for Real-Time Non-invasive Neuromodulation *in vivo*

**DOI:** 10.3389/fnins.2020.00445

**Published:** 2020-05-12

**Authors:** Huifang Yang, Yi Yuan, Xingran Wang, Xin Li

**Affiliations:** Institute of Electrical Engineering, Yanshan University, Qinhuangdao, China

**Keywords:** CLTUS, real-time, neural oscillation, TLE, neuromodulation

## Abstract

The closed-loop brain stimulation technique plays a key role in neural network information processing and therapies of neurological diseases. Transcranial ultrasound stimulation (TUS) is an established neuromodulation method for the neural oscillation in animals or human. All available TUS systems provide brain stimulation in an open-loop pattern. In this study, we developed a closed-loop transcranial ultrasound stimulation (CLTUS) system for real-time non-invasive neuromodulation *in vivo*. We used the CLTUS system to modulate the neural activities of the hippocampus of a wild-type mouse based on the phase of the theta rhythm recorded at the ultrasound-targeted location. In addition, we modulated the hippocampus of a temporal lobe epilepsy (TLE) mouse. The ultrasound stimulation increased the absolute power and reduced the relative power of the theta rhythm, which were independent of the specific phase of the theta rhythm. Compared with those of a sham stimulation, the latency of epileptic seizures was significantly increased, while the epileptic seizure duration was significantly decreased under the CLTUS. The above results indicate that the CLTUS can be used to not only modulate the neural oscillation through the theta-phase-specific manipulation of the hippocampus but also effectively inhibit the seizure of a TLE mouse in time. CLTUS has large application potentials for the understanding of the causal relationship of neural circuits as well as for timely, effective, and non-invasive therapies of neurological diseases such as epilepsy and Parkinson’s disease.

## Introduction

In recent years, closed-loop brain stimulation techniques, such as the deep brain, optogenetic, and transcranial magnetic stimulationshave rapidly developed (Kraus et al., 2016; Milosevic et al., 2018; Zhang Z. et al., 2018). Compared with open-loop brain stimulation, closed-loop brain stimulation can perform brain neuromodulation through the brain state in real time and achieve the targeted on-demand stimulation (Müller et al., 2012; Cagnan et al., 2017; Zhou et al., 2019). Studies on memory and learning mechanisms demonstrated that stimulations of the brain tissue at specific phases of the neural oscillation produce different effects, such as the long-term potentiation or long-term inhibition (Hyman et al., 2003; Ezzyat et al., 2018). In addition, in a therapy against epilepsy, it is necessary to carry out stimulation at the beginning of seizures, which can not only significantly reduce the amount of stimulation, but also improve the stimulation effect (Berényi et al., 2012; Esther et al., 2013; Liu et al., 2018). Closed-loop brain neuromodulation techniques are increasingly used for the investigation of neural network information processing and treatments of various neurological and neuropsychiatric disorders.

Transcranial ultrasound stimulation (TUS) is a physical neuromodulation technique, which is non-invasive and has a large penetration depth and high spatial resolution (Bystritsky et al., 2011; Fomenko et al., 2018). TUS has attracted great attention and has been used in a large number of animal and human experiments (Bystritsky and Korb, 2015; Niu et al., 2018; Yu et al., 2019). Hippocampus that is closely related to learning and memory mechanisms in mice/rats can modulate and encode neural information. Previous studies have found that TUS can modulate neural activity of hippocampus, enhance the amplitude of the local field potential (LFP) and multi-unit activity, increase and induce band oscillations accompanying the increase in phase-amplitude coupling between low- and high-frequency bands (Tufail et al., 2010; Yuan et al., 2016). In addition, previous studies also found that TUS has positive therapeutic effects on neurological diseases. For example, TUS applied to a mouse/rat epilepsy model led to fewer spontaneous recurrent seizures and depression in the chronic period of epilepsy, and increased the performances in behavioral tasks assessing the sociability and interval between seizures (Min et al., 2011; Hakimova et al., 2015; Li et al., 2019a, b). Previous studies have reported that transcranial magnetic stimulation and transcranial direct-current stimulation can non-invasively and effectively inhibit epileptic seizures (Kimiskidis et al., 2014; San-Juan et al., 2015). However, compared to TUS, their spatial resolution and penetration depth are lower (Bystritsky et al., 2011).

However, all TUS systems provide brain stimulation in an open-loop pattern, which sends out stimulus signals at artificially prescribed times and cannot automatically generate stimuli according to the changes in physiological signals of the brain tissue. A closed-loop transcranial ultrasound stimulation (CLTUS) system would be of significance to reveal the neural information processing mechanism and optimize the treatments of neurological diseases. In this study, we developed a CLTUS system for real-time non-invasive neuromodulation *in vivo*. CLTUS was used to modulate the neural activities based on the theta phase locally recorded by using a recording electrode implanted in the mouse hippocampus CA1. The absolute and relative powers of the theta rhythm were calculated before and after CLTUSs at different theta phases. Furthermore, we used CLTUS to inhibit the mouse temporal lobe epilepsy (TLE) seizure in real time by the online detection of epileptic spikes. The latency duration of epileptic seizures and epileptic seizure duration were calculated to evaluate the inhibition effect of CLTUS on the TLE.

## Materials and Methods

### Animals and Operation

A total of 11 C57BL/6 wild-type mice were used in the experiment (all male, body masses: 20–25 g, 4–5-weeks-old, Beijing Vital River Laboratory Animal Technology Co., Ltd., China). Our study protocols were submitted to and approved by the Animal Ethics and Administrative Council of Yanshan University. A closed-loop animal-temperature-maintenance instrument (69002, Ruiwode, Shenzhen, China) was used to maintain the body temperature at ∼37°C during all experiments. During the operation, the mice were anesthetized with 2% isoflurane and fixed on the adapter after losing their mobility. (i) In an experiment on the modulation of the neural oscillation, a tungsten microelectrode (WE50030.1B10, MicroProbe, United States) was implanted into the hippocampal CA1 region of the mouse [*N* = 6, anterior–posterior (AP) = −2 mm, medial–lateral (ML) = 2 mm, and dorsal–ventral (DV) = 1.5 mm relative to the bregma]. (ii) In an experiment on the modulation of the TLE, kainic acid (KA) (1 μg/1 μL in saline, Tocris, United States) was unilaterally microinfused into the CA3 area (AP = −2.0 mm, ML = −2.3 mm, and DV = 2.0 mm from the bregma) through a needle (33 gauge, NanoFil, World Precision Instruments, United States) connected to a 10-μL Hamilton syringe. The flow rate (0.05 μL min^–1^) was regulated by a syringe pump (SP101i, World Precision Instruments, United States). After the injection, we left the needle for 5 min to prevent leakage before slowly pulling it out. A tungsten microelectrode (WE50030.1B10, MicroProbe, United States) was inserted into the CA3 region (*N* = 5, AP = −2.0 mm, ML = −2.3 mm, and DV = 2.0 mm from the bregma) 2 h after the generation of the TLE model. In all experiments, reference and ground screw electrodes were inserted into the nasal bone. All mice were anesthetized with 0.3% isoflurane during the CLTUS experiments.

### Ultrasound System and Parameters

The ultrasound stimulation system used in our previous study (Wang et al., 2019) was also used in this study. A focused ultrasound transducer (V301-SU, focus, diameter: 25.4 mm, radius of curvature: 40 mm, Olympus, United States) with a collimator was used to align the hippocampal region so that the triggered ultrasound wave can reach the targeted region. The ultrasound parameters for the experiment on the modulation of the neural oscillation included a fundamental frequency of 500 kHz, pulsed repetition frequency of 1 kHz, stimulation duration of 400 ms, and duty cycle of 40%, while those for the experiment on the modulation of the TLE were 500 kHz, 500 Hz, 30 s, and 5%, respectively. In a sham stimulation (sham-stim), we turned off the amplifier, while the other experimental procedures were the same as those for the CLTUS. The CLTUS and sham-stim were performed in a random order. In all experiments, both pulsed and continuous ultrasound pressures were ∼0.23 MPa, corresponding to *I*_sppa_ of 1.75 W/cm^2^. The *I*_spta_ values corresponding to *I*_sppa_^∗^ (duty cycle) were 700 mW/cm^2^ for the modulation of the neural oscillation and 66.5 mW/cm^2^ for the inhibition of epilepsy.

### CLTUS Modulation of the Neural Oscillation

**FIGURE 1:**
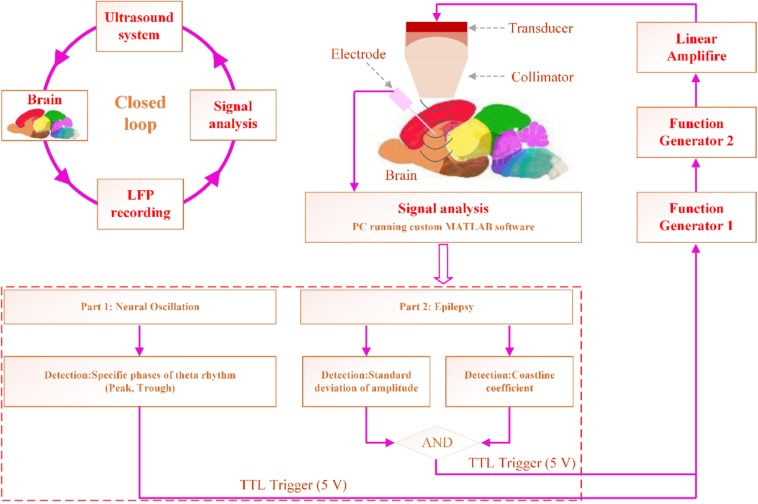
CLTUS system design. In the modulation experiment, the specific phase of the theta rhythm is detected online. In the experiment on the inhibition of the seizure of TLE, the seizure is detected online by calculating the standard deviation of the amplitude and CL. Based on the detection results, the computer sends a TTL high-level signal, which triggers the function generator to send a signal. The signal is sent to the amplifier, which drives the ultrasound transducer to emit an ultrasound wave for CLTUS.

We used the reported method (Feng et al., 2012) to record raw LFP data (time interval: 1 s) in the buffer area, analyze the data to predict the next peak or trough. The Hamming window is used to design the band-pass filter with a frequency range of 4–12 Hz and transition frequency of 4 Hz. We set the period threshold in the range of 0.08–0.25 s, calculate the threshold of the amplitude of the theta rhythm, and find the locations of all minimum and maximum values of the theta waveform. Subsequently, we calculate the average period (*A*_p_) and peak-to-peak value. Finally, we predict the time of the next peak (*t*_p_ = *A*_p_ – *t*_*c*_ – *t*_peak_) or trough (*t*_*r*_ = *A*_p_ – *t*_*c*_ – *t*_trough_), where *t*_*c*_ is the time interval from the acquisition of the data to the determination of the theta rhythm, which can be calculated by the MATLAB clock function, and *t*_peak_ and *t*_trough_ are the times of the last peak and trough of the cache data, respectively. As shown in Figure 1, when the specific phase of the theta rhythm is predicted, the computer sends a transistor–transistor logic (TTL) high-level signal, which triggers the function generator to send a signal. The signal is sent to the amplifier, which drives the ultrasound transducer to emit an ultrasound wave for CLTUS.

### CLTUS for Inhibiting of the Seizure of a TLE Mouse

We calculated the amplitude standard deviation (Hjorth, 1970) and coastline coefficient (CL) (Korn et al., 1987) of the LFP signals from the hippocampus. The standard deviation of the amplitude reflects the activity of the LFP signal and is widely used in the detection of seizures (Hjorth, 1970). It can be calculated as

(1) $$\text{m}=\frac{1}{N}\,\sum_{i=1}^{N}x\,\left(i\right),H=\sqrt{\frac{1}{N}\,\sum_{i=1}^{N}{\left(x\,\left(i\right)-m\right)}^{2}},$$

where *H* is the standard deviation of the signal amplitude, which reflects the smoothness of the signal. *H* increases upon a considerable change in amplitude of the signal.

The CL describes the characteristics of the signal waveform variation by accumulating the amplitude difference between two adjacent points of the calculation signal (Korn et al., 1987). It can be calculated as

(2) $$C\,L=\frac{1}{N-1}\,\sum_{i=1}^{N}\left|x\,\left(i\right)-x\,\left(i+1\right)\right|,$$

where *N* is the length of the data. The CL increases when the waveform rapidly changes.

We recorded the LFP signals from the hippocampus and simultaneously calculated H and CL of the LFP signals online. The H and CL of the LFP without seizure of epilepsy were calculated as a baseline. The value of H three times (or more) than that of the baseline and CL value two times (or more) that of the baseline were set as the condition of seizure. As shown in Figure 1, when both values satisfied the set conditions of the epileptic waveform, the computer sends a transistor–transistor logic (TTL) high-level signal to perform CLTUS.

### Calculation of the Power Spectrum

We analyzed the absolute (AP) and relative (RP) powers of the theta rhythm [4–12 Hz] induced by the CLTUS according to our previous study (Wang et al., 2019). The total AP of the frequency bands was obtained by summing the APs of the frequency bands in the range of 1–200 Hz. The RP of the theta rhythm was equal to the corresponding AP of the rhythm divided by the total AP. The time–frequency diagram was calculated using short-time Fourier transforms with a Hamming window, whose length was 1000 points. The mean absolute power (MAP) and mean relative power (MRP) of the theta rhythm before and after the CLTUS were calculated by

(3) $$M\,A\,P=\sum_{T_{1}}^{T_{2}}\left|A\,P\right|\,/\,\left(T_{2}-T_{1}\right),M\,R\,P=\sum_{T_{1}}^{T_{2}}R\,P\,/\,\left(T_{2}-T_{1}\right),$$

where *T*_1_ and *T*_2_ are the start and end times of the theta rhythm, respectively. The percent changes in MAP and MRP were calculated as

(4) $$\Delta \,A\,P/A\,P=\left(M\,A\,P_{0-3\,s}-M\,A\,P_{-1-0\,s}\right)/M\,A\,P_{-1-0\,s},$$

(5) $$\Delta \,R\,P/R\,P=\left(M\,R\,P_{0-3\,s}-M\,R\,P_{-1-0\,s}\right)/M\,R\,P_{-1-0\,s},$$

where MAP_0__–__3__s_, MRP_0__–__3__s_, MAP_–__1__–__0__s_, and MRP_–__1__–__0__s_ are the MAPs and MRPs in the ranges of 0–3 and −1 to 0 s, respectively.

### Statistical Analysis

The results for the states at different times were evaluated with a one-way analysis of variance (ANOVA). Differences were considered significant when *p* < 0.05. The statistical analyses were carried out using MATLAB.

## Results

### CLTUS Modulation of the Neural Oscillation Based on the Specific Phase of the Theta Rhythm

**FIGURE 2:**
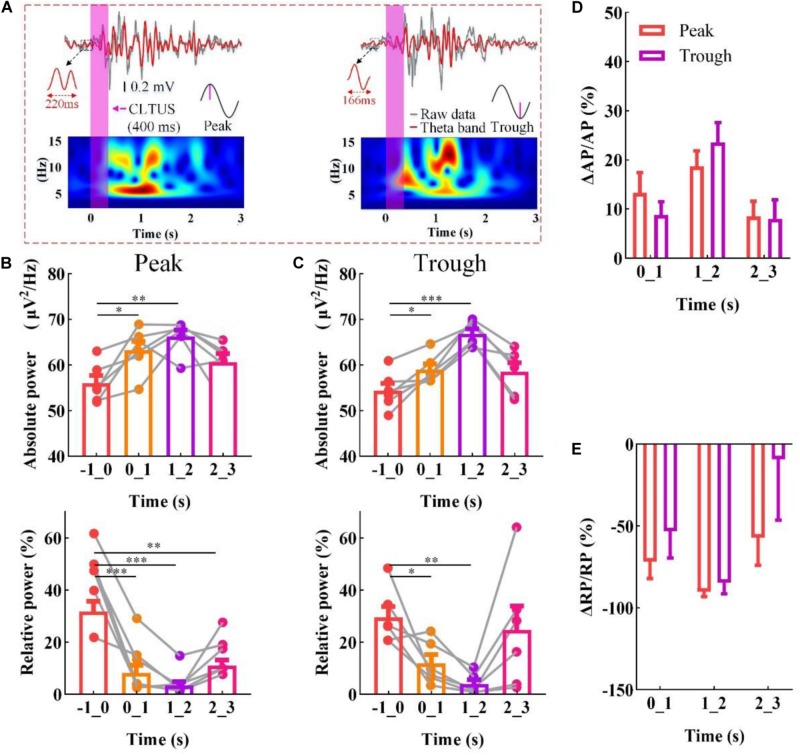
CLTUS for neural oscillation based on the specific phase of the theta rhythm. **(A)** (top) LFP signals and different phases of the theta rhythm (peak and trough); (bottom) time–frequency diagram of LFP signals (4–16 Hz). **(B,C)** (top) MAP of the theta rhythm (peak and trough, respectively *p < 0.05, **p < 0.01, ***p < 0.001, *N* = 6, mean ± SEM, one-way ANOVA); (bottom) RP of the theta rhythm (peak and trough, respectively, *p < 0.05, **p < 0.01, ***p < 0.001, *N* = 6, mean ± SEM, one-way ANOVA). Changes in **(D)** AP (ΔAP/AP) and **(E)** RP (ΔRP/RP) of the theta rhythm upon the CLTUS.

The theta rhythm is one of the most prominent rhythms in the mammalian brain (György, 2002; Colgin, 2013; György et al., 2013) and is crucial in the evaluation of the effect of an external stimulation (Klemm, 1972; Llinás et al., 2005; Gregor et al., 2012). To verify that the CLTUS can modulate the neural oscillation in time, we used it to stimulate the CA1 region of the mouse according to the specific phase of the theta rhythm (peak or trough). Figure 2A shows the LFP signals, corresponding theta rhythm, and time-frequency diagram at the peak and trough of the theta rhythm. The results indicate that the amplitude of the LFP and amplitude and power intensity of the theta rhythm are significantly increased after the CLTUSs at the two states. This could be explained as the ultrasound stimulated the activity of the neurons and induced discharge of a large number of neurons, which led to a high-amplitude theta wave. With the recovery of the neuronal activity, the amplitude of the theta wave gradually decreases and tend to return to the level before the CLTUS. Further, we calculate the power spectra of the theta rhythm at different times. We use −1 to 0 s as the time interval starting 1 s before the start of the stimulus and 0–1, 1–2, and 2–3 s as the time intervals up to 3 s after the end of the stimulus. As shown in Figures 2B,C (top), the MAP of the theta rhythm is significantly increased after the CLTUS, and then gradually returned to the level before the CLTUS. These results indicate that the CLTUSs at the peak and trough altered the MAP of the theta rhythm in the hippocampus (*N* = 6, mean ± standard error of the mean (SEM), ^∗^*p* < 0.05, ^∗∗^*p* < 0.01, ^∗∗∗^*p* < 0.001, one-way ANOVA). Furthermore, we investigate whether the CLTUS altered the RP of the theta rhythm. The experimental results are shown in Figures 2B,C (bottom). Notably, unlike the AP of the theta rhythm, its RP is significantly decreased after the CLTUS at the peak and trough, and then gradually increased (*N* = 6, mean ± SEM, ^∗^*p* < 0.05, ^∗∗^*p* < 0.01, ^∗∗∗^*p* < 0.001, one-way ANOVA). To evaluate the dependences of the changes in theta rhythm induced by the CLTUSs at the peak and trough, we analyze the changes in AP (ΔAP/AP) and RP (ΔRP/RP) of the theta rhythm. As shown in Figures 2D,E, no significant differences in ΔAP/AP and ΔRP/RP were observed between the CLTUSs at the peak and trough of the theta rhythm. The above results show that the CLTUS at the specific phase can increase the AP of the theta rhythm and reduce its RP. In addition, the stimulation effect is independent on the specific phase (peak or trough) of the theta rhythm.

### Inhibition of the Seizure of a TLE Mouse by CLTUS

**FIGURE 3:**
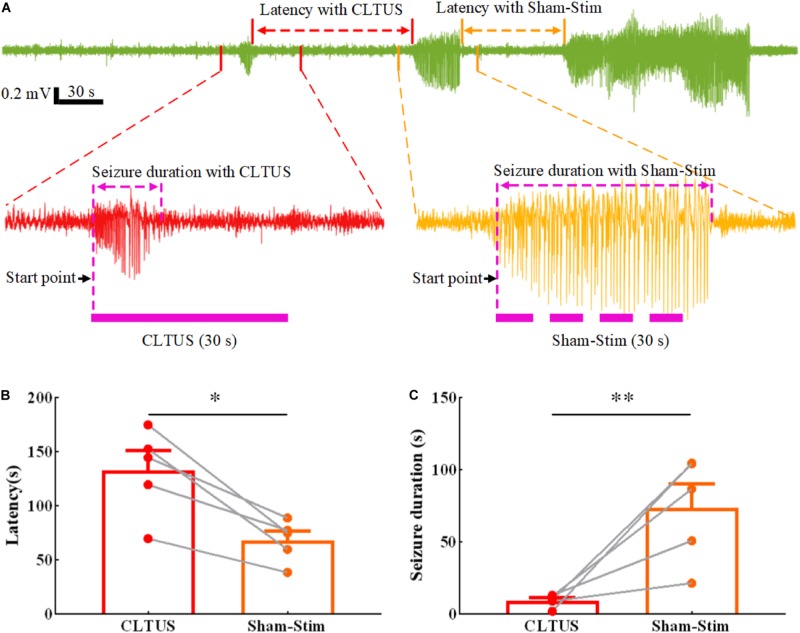
Inhibition of the seizure of a TLE mouse in real time by CLTUS. **(A)** Epileptic states of a typical mouse under CLTUS and sham-stim. **(B)** Latencies of epileptic seizure under CLTUS and sham-stim (CLTUS: 132.8 ± 18.0 s, sham-stim: 74.2 ± 11.5 s, *p < 0.05, *N* = 5, mean ± SEM, one-way ANOVA). **(C)** Durations of epileptic seizure under CLTUS and sham-stim (CLTUS: 9.3 ± 1.9 s, sham-stim: 73.8 ± 16.3 s, **p < 0.01, *N* = 5, mean ± SEM, one-way ANOVA).

Temporal lobe epilepsy with the characteristics of frequent seizures and difficult diagnosis and treatment is the most common epileptic syndrome in adults (Saling, 2009; De et al., 2011; Monti and Meletti, 2015). To verify that the CLTUS can effectively modulate the neurological disease with a neuronal abnormal discharge in time, we recorded the data of TLE for 60 min and simultaneously carried out CLTUS on the TLE mouse model. In order to achieve self-control, we used the same animal for CLTUS and sham-stim. Figure 3A shows the epileptic states of a typical mouse under CLTUS and sham-stim. The experimental results show that our method can detect epilepsy online and supply the ultrasound stimulation based on the detected results. In addition, the latency of epileptic seizures was significantly increased, while the epileptic seizure duration was significantly decreased under the CLTUS. To quantitatively evaluate the effect of the CLTUS on the inhibition of the epileptic seizures, we analyzed the experimental data for five TLE mice. As shown in Figures 3B,C, the latency of epileptic seizures under the CLTUS was significantly larger than that under sham-stim (CLTUS: 132.8 ± 18.0 s, sham-stim: 74.2 ± 11.5 s, ^∗^*p* < 0.05, *N* = 5, mean ± SEM, one-way ANOVA). The epileptic seizure duration under the CLTUS was significantly smaller than that under sham-stim (CLTUS: 9.3 ± 1.9 s, sham-stim: 73.8 ± 16.3 s, *N* = 5, mean ± SEM, ^∗∗^*p* < 0.01, one-way ANOVA). These results indicate that the CLTUS can effectively inhibit the seizure of TLE in time.

## Discussion

In this study, we developed a CLTUS system for real-time non-invasive neuromodulation *in vivo*. The CLTUS system was used to modulate the neural oscillations in CA1 of the mouse according to the specific phase of the theta rhythm (peak or trough). The experimental results show that the system can modulate the neural activity in real time according to the theta rhythm. The AP of the theta rhythm was increased, while its RP was decreased after the CLTUS at the peak or trough of the theta rhythm. In addition, the RP and AP of the theta rhythm were independent on the specific phase under the CLTUS. Furthermore, we carried out CLTUS on the TLE mouse model. The system could accurately detect the seizures of epilepsy and supply real-time CLTUS according to the detected results. The experimental results also showed that the CLTUS could effectively inhibit the seizures, increase the latency of epilepsy, and reduce the seizure duration of epilepsy. The results indicate that the CLTUS can be used to modulate the neural oscillations and effectively inhibit the seizure of TLE in time. To the best of our knowledge, this is the first report on the development of a CLTUS system for the temporal, effective, and non-invasive neuromodulation *in vivo*. It could be potentially used in the analyses of neural information processing mechanisms and optimization of the treatments of neurological diseases such as epilepsy and Parkinson’s disease.

Compared to open-loop ultrasound stimulation, CLTUS performed ultrasound stimulation at a specific time according to the state of brain neural activity to achieve timely and effective neuromodulation. In addition, open-loop ultrasound stimulation is not universally applicable to the treatment of epilepsy, because it does not provide corresponding stimulation according to epileptic seizure. The CLTUS can be used for stimulating the epilepsy according to the time of epileptic seizure, which makes the stimulation scheme more reasonable and has better general applicability.

Further, we compare our method with other closed-loop brain stimulation techniques such as the deep brain and optogenetic stimulations. Closed-loop deep brain stimulation can effectively inhibit the abnormal discharge in real time and thus is widely used in the treatments of epilepsy and Parkinson’s disease (Hamani et al., 2009; Rosin et al., 2011). Compared with closed-loop deep brain stimulation, the CLTUS is a non-invasive brain stimulation technique, which does not require surgery for the implantation of the electrode in the brain tissue. Closed-loop optogenetic stimulation with a precise targeting and high temporal and spatial resolutions is widely used in studies on neural circuits and disease models (Paz et al., 2013; Grosenick et al., 2015). Compared with closed-loop optogenetic stimulation, the CLTUS is a label-free non-invasive neuromodulation technique and thus can be used for human brain stimulation. As the electrodes and fibers are directly implanted into the targeted area in the deep brain and optogenetic stimulation methods, their stimulus accuracies are higher than that of the CLTUS.

Safety is a key indicator in the evaluation of the CLTUS for the modulation of brain tissues. The ultrasound intensities (*I*_spta_) used in our experiments were ∼533 mW/cm^2^ (for the modulation of the neural oscillation) and ∼66.5 mW/cm^2^ (for the inhibition of epilepsy), considerably smaller than the upper regulatory limit for non-obstetric ultrasound imaging [720 mW/cm^2^ (FDA, 2018)]. The mechanical index of our experiment was 0.28, which is within the range of safety guidelines [∼1.9 (Lee, 1998)]. All ultrasound parameters used in our CLTUS experiments are within the ranges of safety guidelines for clinical ultrasound imaging.

Recently, Sato et al. (2018) and Guo et al. (2018) reported that ultrasound stimulation activates cortical neurons by non-specific auditory responses. However, these results are countered in a recent report by Mohammadjavadi et al. (2019), who demonstrated that the direct activation of central motor neural circuits occurs through ultrasound stimulation rather than through auditory responses (fundamental frequency: 500 kHz, pulsed repetition frequency: 1.5 kHz, duty cycle: 80%, *I*_spta_: 2.9 W/cm^2^). As *I*_spta_ used in our study is lower than that in the study by Mohammadjavadi et al. (2019), we believe that our study demonstrates directly ultrasound mediated neural oscillation and inhibition of epileptic seizure.

In previous studies, open-loop low-intensity ultrasounds were used to stimulate non-human primates from the cortex (i.e., visual cortex) to the deep brain area (i.e., amygdala) (Deffieux et al., 2013; Wattiez et al., 2017; Folloni et al., 2019; Lennart et al., 2019). They demonstrated that the open-loop ultrasound stimulation can modulate the brain neural network. It was also used to stimulate humans from the cortex (i.e., somatosensory cortex) to the deep brain area (i.e., thalamus) (Panczykowski et al., 2014; Monti et al., 2016). The open-loop ultrasound stimulation could modulate the neural activity. In addition, the disturbance of consciousness after a severe brain injury could be treated through the stimulation of the human thalamus. The above studies indicate that the ultrasound stimulation can provide the non-invasive modulation of neural activities with a large penetration depth (to the human thalamus, ∼ 7 cm). In addition, the ultrasound had good therapeutic and protective effects on neurological diseases (Huang et al., 2017; Li et al., 2017; Liu et al., 2017; Su et al., 2017a, b; Chen et al., 2018; Zhang D. et al., 2018). Considering its large penetration depth and stimulation effect on neural oscillations and neurological diseases, as well as the timely, effective, and on-demand characteristics, we believe that the CLTUS has good application prospects in neural information processing and treatments of neurological diseases.

We performed only the closed-loop ultrasound stimulation based on the specific phase of the theta rhythm. The modulation effects on different rhythms with specific phases are still unclear. In addition, previous studies have shown that the ultrasound parameters significantly affect the neuromodulation (King et al., 2013; Kim et al., 2014; Yu et al., 2016, 2019). In our experiment, the stimulation parameters were set in advance and were not optimized according to the stimulation effect. We did not perform CLTUS under the optimal parameters to obtain the best effects of neurological modulation and inhibition of epilepsy. Considering these limitations, we will continue to carry out in-depth studies and more experiments to stimulate brain tissues based on the specific phases of different rhythms and analyze optimal ultrasound stimulation parameters.

In summary, we developed a CLTUS system for real-time non-invasive neuromodulation *in vivo*. It was used to not only modulate the neural oscillation according to the specific phase of the theta rhythm but also effectively inhibit the seizure of a TLE mouse model in real time. The CLTUS has large application potentials for the understanding of the causal relationship of neural circuits and timely, effective, and non-invasive therapies of neurological diseases such as epilepsy and Parkinson’s disease.

## Data Availability Statement

The raw data supporting the conclusions of this article will be made available by the authors, without undue reservation, to any qualified researcher.

## Ethics Statement

The animal study was reviewed and approved by the Animal Ethics and Administrative Council of Yanshan University.

## Author Contributions

XL and YY designed and coordinated the study. All authors carried out the experiment and data process, drafted the manuscript, and gave final approval for publication.

## Conflict of Interest

The authors declare that the research was conducted in the absence of any commercial or financial relationships that could be construed as a potential conflict of interest.
